# The GH/IGF-1 Axis and Heart Failure

**DOI:** 10.2174/157340309788970306

**Published:** 2009-08

**Authors:** Graziella Castellano, Flora Affuso, Pasquale Di Conza, Serafino Fazio

**Affiliations:** Department of Internal Medicine, School of Medicine, University of Naples “Federico II”, Naples, Italy

**Keywords:** GH/IGF-1 axis, Chronic heart failure, Acromegaly, GH deficiency.

## Abstract

The growth hormone (GH)/insulin-like growth factor 1 (IGF-1) axis regulates cardiac growth, stimulates myocardial contractility and influences the vascular system. The GH/IGF-1 axis controls intrinsic cardiac contractility by enhancing the intracellular calcium availability and regulating expression of contractile proteins; stimulates cardiac growth, by increasing protein synthesis; modifies systemic vascular resistance, by activating the nitric oxide system and regulating non-endothelial-dependent actions. The relationship between the GH/IGF-1 axis and the cardiovascular system has been extensively demonstrated in numerous experimental studies and confirmed by the cardiac derangements secondary to both GH excess and deficiency. Several years ago, a clinical non-blinded study showed, in seven patients with idiopathic dilated cardiomyopathy and chronic heart failure (CHF), a significant improvement in cardiac function and structure after three months of treatment with recombinant GH plus standard therapy for heart failure. More recent studies, including a small double-blind placebo-controlled study on GH effects on exercise tolerance and cardiopulmonary performance, have shown that GH benefits patients with CHF secondary to both ischemic and idiopathic dilated cardiomyopathy. However, conflicting results emerge from other placebo-controlled trials. These discordant findings may be explained by the degree of CHF-associated GH resistance. In conclusion, we believe that more clinical and experimental studies are necessary to exactly understand the mechanisms that determine the variable sensitivity to GH and its positive effects in the failing heart.

## INTRODUCTION

Growth hormone (GH), a 191 amino acid single-chain peptide, is synthesized and secreted by the somatotroph cells of the anterior pituitary gland [1, 2]. Its secretion is strictly regulated by two hypothalamic neurohormones: GH releasing factor (GHRF) and GH inhibiting factor (somatostatin). The ratio between these two factors represents the mechanism by which neurologic and extra-neurologic influences may functionally affect GH release [2-9]. Furthermore, GH can modulate its own secretion by different feedback loops: indirectly by producing insulin-like growth factor-1 (IGF-1), which inhibits somatotroph cells and stimulates somatostatin release, or directly by inhibiting GHRF messenger RNA (mRNA) and by stimulating somatostatin mRNA synthesis [10].

GH secretion is pulsatile, and is regulated by a number of neurologic, metabolic and hormonal influences: during most of the day, the plasma GH level of adults is 5 ng/ml, with one or two sharp spikes three to four hours after meals. The lowest circulating level is early in the morning and highest about one hour after the onset of deep sleep [11-14]. Secretion is enhanced by α_2_ agonists, hypoglycemia and daily life stresses, and inhibited by β and α_1_ agonists, glucocorticoids and aging [12, 14-17].

The biological effects of GH are mediated by the interaction with a specific receptor (GHR), a single chain trans-membrane protein, expressed in almost all cellular types (liver membranes, adipocytes, fibroblasts, lymphocytes, myocytes) [8, 11, 18,  19]. Its dimerization activates the Jak/Stat pathway (Janus Kinase and Signaling Transducer and Activates of Transcription), which induces intracellular signal transduction, thereby altering calcium (Ca^2+^) trafficking, regulating expression of contractile and cytoskeletal proteins and modifying activation of intrinsic neurohormonal networks [20].

GH exerts its effects either directly or indirectly [2, 21, 22]. Most of the indirect effects are mediated by induction of IGF-1 expression in the liver and in peripheral tissues [23-27]. It is well known that IGF-1 is the principal, but not the only, GH mediator. For instance, GH stimulates induction of c-myc proto-oncogene in various tissues and of platelet-derived growth factor in the heart [28,  29]. But the role of these and other growth factors is still unknown.

IGF-1, a 70 amino acid single chain protein, structurally homologous to pro-insulin, is synthesized in liver and kidney, although the local production in other tissues appears to be important in mediating, by paracrine or autocrine mechanisms, GH anabolic and growth-promoting effects [30-33]. IGF-1 circulates bound to protein carriers (IGFBPs), which serve not only to transport IGF-1 in the circulation but also to prolong its half life, modulate its tissue specificity and strengthen or neutralize its biological actions [31]. The serum concentration of IGFBPs is influenced by circulating GH levels, but does not have a circadian pattern. The intracellular signal pathways involved in IGF-1 transduction implicate insulin receptor substrate (IRS)-1, phosphatidylinositol (PI) 3-kinase, phospholipase C (PLC)-g1, mitogen-activated protein kinase (MAPK) and extracellular signal-regulated kinase (ERK) cascade [34].

The diminished age-related amplitude of GH pulses and the increased resistance to GH action contribute to reduce IGF-1 plasma concentration. The mechanisms underlying these age-related modifications include peripheral influences (gonadal steroids, adiposity), changes in hypotalamic neuropeptides and neurotransmitters, and increase in somatostatin secrection. [35]. Although the decline of GH/IGF-1 axis is associated with an increase in GH/IGF-1 receptors on cardiomyocytes, this increase fails to compensate the reduction of GH secretion probably because of the diminished intracellular signal transduction [36, 37]. In fact, in rodents, it has been widely demonstrated that with aging there is a reduction of JAK2 phosphorilation, a decline of MAP kinase activity, a reduction of STAT3 activation and a decrease in nuclear translocation [37-40]. GH/IGF-1 deficiency contributes to physiological age-related cardiovascular modifications, such as decrease in the number of cardiomyocytes [41-43], rarefaction of coronary arterioles [44], increase in fibrosis and collagen deposition [45-48], reduced protein synthesis [49] and alteration of contractile proteins with reduction in myosin-actin bridges [50].

## PHYSIOLOGICAL EFFECTS OF GH

GH alters body’s homeostasis and its effects can generally be described as anabolic. GH directly stimulates chondrocyte division and multiplication; it increases calcium retention, thereby strengthening bone mineralization [51]; promotes lipolysis and protein synthesis, by stimulating amino acid uptake [16, 52-55]; induces hyperglycemia, consequent to insulin resistance and gluconeogenesis [52, 56]; increases muscle mass, through sarcomere hyperplasia, and stimulates the immune system. In addition, GH increases peripheral conversion of thyroid hormone thyroxine (T4) to triiodothyronine, with a consequent decline of thyroid stimulating hormone and T4 levels [57]. It also activates the renin-angiotensin-aldosterone system and decreases atrial natriuretic peptide circulating level [58, 59].

## CARDIOVASCULAR EFFECTS

**Fig. (1):**
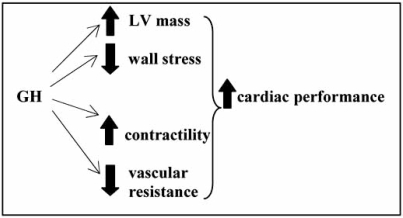
Growth hormone (GH), by increasing left ventricle mass and myocardial contractility, and decreasing wall stress and vascular resistance, enhances cardiac performance.

Besides growth promoting and metabolic effects, the GH/IGF-1 axis regulates cardiac growth, stimulates myocardial contractility and influences the vascular system (Fig. **1**).

The myocardium and the endothelium not only express receptors for both GH and IGF-1, but also produce IGF-1 locally. Thus, there is a direct action of GH by endocrine mechanism and/or indirect action by autocrine/paracrine mechanisms of IGF-1 [30-32]. On vascular system, the GH/IGF-1 axis exerts its effects by activating the nitric oxide (NO) system and regulating non-endothelial-dependent actions [60-69]. NO production relaxes arterial smooth muscle cells, thereby reducing vascular tone. Furthermore, NO inhibits proliferation and migration of smooth muscle cells, reduces platelet adhesion, decreases lipoxygenase activity and oxidized LDL-cholesterol [60-69]. Recently, NO has been shown to modulate cardiac cytoskeletal functions by altering calcium myofilament responsiveness [70]. In addition, IGF-1 may cause vasorelaxation both by enhancing Na^+^/K^+^ ATPase activity [71] and regulating gene expression of K_ATP_ channel in vascular smooth cells [72]. This ATP-sensitive potassium channel consists of two subunits: the sulfonylurea receptor and the inwardly rectifying potassium channel, which could be critical in regulating vascular tone [73, 74].

The GH/IGF-1 axis may also regulate cardiac growth and metabolism, by increasing amino acid uptake, protein synthesis, cardiomyocyte size and muscle-specific gene expression. Specifically IGF-1 promotes cardiac hypertrophy and increases muscle specific gene transcription (namely, troponin I, myosin light chain-2, and α-actin) [75-77]. Moreover, IGF-1 promotes collagen synthesis by fibroblasts, whereas GH increases the collagen deposition rate in the heart [78-81]. Substantial evidence indicates that IGF-1 influences the trophic status of myocardium by reducing apoptosis of cardiomyocytes, thus preventing myocyte loss [76, 82].

The GH/IGF-1 axis can also control intrinsic cardiac contractility through different mechanisms: by enhancing myofilament calcium sensitivity [76, 77, 82, 83], modifying intracellular calcium transient through an increase in L- type calcium channel activity [84, 85] and up-regulating sarcoplasmatic reticulum ATPase (SERCA) levels [86, 87]. SERCA up-regulation may cause an increase in contractility, enhancing calcium contractile reserve in the sarcoplasmatic reticulum and allowing a higher calcium peak level on stimulation.

While IGF-1 positively affects cardiac contractility, GH physiological role, although GHRs are expressed on the heart, probably does not include acute modulation of myocardial contractility, but it needs to mediate some other functions such as protein synthesis or local IGF-1 production [82, 88].

Moreover, GH induces myosin phenoconversion toward the low ATPase activity V3 isoform. The prevalence of V3 isoform increases the number of actin-myosin cross-bridges and their attachment time, enhances protein calcium sensitivity and calcium availability and allows the myocardium to function at lower energy cost [76, 77]. V3 isoform also prevails in pathologic cardiac hypertrophy secondary to hemodynamic overload, to compensate depressed contractility and high wall stress.

Although GH reduces energy output, it favours the conversion of metabolic energy to external work and enhances the intrinsic ability of the myofilament to develop force, resulting in an improvement of LV performance [89]. In conclusion, GH improves myocardial energy metabolism reducing oxygen consumption and energy demand, even in failing heart in which the increment in wall stress increases oxygen demand [90]

The relationship between the GH/IGF-1 axis and the cardiovascular system has been extensively demonstrated in numerous experimental studies and confirmed by the derangements of cardiac structure and function reported in patients with both GH excess (acromegaly) and GH deficiency (GHD).

## CLINICAL EVIDENCE OF GH EXCESS IN HUMANS

Acromegaly is a clinical condition consequent to chronic GH excess that affects the heart. Acromegalic cardiac involvement was first described by Huchard in 1895 [91]. Subsequent reports documented that chronic GH excess leads to cardiac functional and morphological abnormalities [30, 76, 77, 92-97].

Acromegalic cardiomyopathy can be divided into three main stages [22, 89]. The early stage is characterized by functional abnormalities: enhanced myocardial contractility, decreased peripheral vascular resistance and increased cardiac output (hyperkinetic state) [22, 89, 98, 99]. In stage 1, ventricular wall thickening is not associated with cavity dilatation, so that relative wall thickness (left ventricular [LV] wall thickness/LV radius) increases and causes a reduction in wall stress and an increase in cardiac performance, according to the Laplace’s law (wall stress=LV pressure/LV relative wall thickness) [10, 22, 89, 99-106]. In this stage, the reduction of wall stress together with the positive effects of GH/IGF-1 on myocardial contractility and systemic vascular resistance produces an improvement in cardiac function. Initially, this increase in wall thickness and LV mass has no negative impact on diastolic function [22, 99, 105]. The intermediate stage (after about five years of active disease) is characterized by biventricular hypertrophy, diastolic dysfunction and impaired cardiac performance, which are undetected under resting condition, but appear on effort [22, 89, 103, 104, 107-109]. Hypertrophy, which entails proliferation of myocardial fibrous tissue, leads to progressive interstitial remodelling, which causes inexorable deterioration of cardiac performance. Diastolic abnormallities, which usually anticipate systolic dysfunction, include prolonged isovolumic relaxation time, decreased early-to-late mitral and tricuspid velocity ratio, reduced diastolic filling wave and increased reversal flow during atrial contraction. These alterations result in impaired ventricular relaxation and enhanced ventricular stiffness [22, 29, 104, 106]. In a very late stage, acromegalic cardiomyopathy is characterized by systolic and diastolic dysfunction that can lead to congestive heart failure, often resistant to conventional therapies, increased myocardial mass, marked ventricular cavity dilatation and high peripheral vascular resistance [22, 30, 98]. It also includes cardiac valve disease (mitral and aortic valve regurgitation), coronary artery disease and arrhythmias [110]. The prevalence of these complications is likely to depend on the duration of GH excess. Myocardial hypertrophy and interstitial fibrosis, which increase as the disease progresses, are responsible for myocardial ischemia, consequent to reduced capillary density, and arrhythmias, due to the interference of the pulse propagation process in the myocardium [111]. Electrocardiographic recordings have demonstrated a higher frequency of ectopic beats, paroxysmal atrial fibrillation or supraventricular tachycardia, sick sinus syndrome, ventricular tachycardia and bundle branch block in acromegalic patients as compared with the normal population [112-114].

The most relevant histological abnormalities are interstitial fibrosis, reduced capillary density, increased extracellular collagen deposition, myofibrillar derangement, lymphomononuclear infiltration and myocyte death due to necrosis and apoptosis [22, 110, 115, 116].

GH excess seems to exert different and potentially opposite effects on the heart: it enhances cardiac performance in early-stage acromegaly, whereas it causes cardiac dysfunction in the intermediate-late phase. This apparent discrepancy is easily clarified: a physiological GH level or short-term excess exert positive inotropic effect, whereas by causing morphological and functional adaptive changes, long-term exposure to GH excess induces cardiac dysfunction and progression to heart failure [76, 77, 92, 98, 106, 117, 118].

GH/IGF-1 may cause acromegalic morphological and functional changes either directly by affecting myocyte growth and contractility, or indirectly by affecting peripheral vascular resistance, modifying extracellular volume and neurohormonal activity. Subsequently, with the increase of arterial stiffness due to hypertrophy and fibrosis of the arterial muscular tunica, about 20-50% of acromegalic patients become hypertensive [119]. Experimental studies about the role of the neurohormonal system in the development and progression of acromegalic cardiomyopathy, have produced conflicting results [30, 120-128]. In the late 1970s, it was reported that chronic GH excess, by eliciting sympathetic overactivity, induces myocardial hypertrophy [120]. Only two decades later, it was demonstrated that GH exerts no sympatho-excitatory effects [122, 129]. Recently, it has been shown that in acromegalic cardiomyopathy, in contrast with other conditions of cardiac hypertrophy, there are low B-type natriuretic peptide (BNP) circulating levels and that the normalization of GH/IGF-1 serum concentrations is followed by an increase in BNP levels [130].

There is compelling evidence that IGF-1 is involved in the intricate cascade of events leading to cardiac hypertrophy. In fact, in response to pressure or volume overload, IGF-1 expression increases in parallel to hypertrophy [131, 132]. Moreover, numerous trials have shown that GH suppression, associated with IGF-1 normalization, reduces cardiovascular mortality to that of general population, which supports the concept that cardiac alterations in acromegaly are strictly related to GH/IGF-1 excess [104,133-141]. By normalizing serum GH and IGF-1 values, somatostatin analogues improve diastolic filling parameters (ventricular isovolumic relaxation and early diastolic filling velocity), reduce volume overload and pulmonary and wedge pressures, and enhance cardiac performance [137, 142,  143]. Data on the effectiveness of acromegalic treatment are still conflicting as regards the effects on ventricular hypertrophy. Some studies demonstrate that treatment can reduce LV mass to a normal value [105], whereas others show no significant change or only a small improvement in LV mass [144]. Although it is not yet known whether the acromegalic heart can return to normal condition, the experimental data available indicate that cardiac hypertrophy is reversible and that the reversal may be complete if GH activity is restored to normal level for a sufficient amount of time [135-137, 140, 141, 144-146]. However, it should be noted that the cardiac effects of somatostatin analogues seem to be related not only to the strict biochemical control of acromegaly, but also to the patient's age and the disease duration before starting treatment [22].

## GH DEFICIENCY

Growth hormone deficiency produces different clinical features depending on the time of onset and disease severity and duration [2, 22, 106, 147]. GHD negatively affects cardiovascular function by directly acting on the heart and endothelium; it also acts indirectly by causing insulin resistance, abdominal obesity, hypercoagulability, increase in serum lipids, reduction in exercise performance and pulmonary capacity [148, 149]. GHD patients have increased total body fat, atherothrombotic and proinflammatory abnormalities, dyslipidemia and decreased insulin-stimulated glucose uptake by fat and skeletal muscle [150, 151]. In addition to the cardiovascular risk factors mentioned above, GHD patients have increased vessel intima-media thickness, which is the earliest morphological change in the development of atherosclerosis [149, 152-155]. Patients with GHD are also affected by endothelial dysfunction, reduced NO production, high peripheral vascular resistance and enhanced aorta stiffness [152-157]. Furthermore, GHD affects cardiac size and function, thereby leading to a reduction in both myocardial growth rate and cardiac performance [158, 159]. Cardiac function decreases because of reduced ventricular mass and intrinsic myocardial contractility [160].

Childhood-onset GHD is characterized by cardiac atrophy with a significant reduction in LV mass, relative wall thickness and cavity dimensions, compared with age-, sex- and height-matched controls [158-162]. Moreover, patients are affected by a hypokinetic syndrome, namely, they have a low ejection fraction, low cardiac output and high peripheral vascular resistance [158, 160-163]. These alterations are more pronounced during physical exercise and, besides reducing skeletal muscle mass and strength, they reduce exercise capacity, as shown by subjective symptoms, low values of achieved workload and exercise duration [160, 164-166]. Adult-onset GHD does not feature a reduction in cardiac mass, but only impaired cardiac performance and exercise capacity [165, 167, 168].

Evidence that cardiac alterations in GHD are strictly related to the GH deficiency comes from many GH replacement trials, which taken together show an increase in LV mass and improvement in cardiac performance, diastolic filling and systolic function after GH treatment [158-160, 163, 164, 166, 169-172]. Although some studies have failed to demonstrate an improvement in cardiac structure or function [173, 174], a meta-analysis that included all trials on the effects of GH replacement included in Medline, Biosis and EMBASE from the year of their inception to June 2002, showed positive effects on LV mass, wall thickness, LV end-diastolic and end-systolic diameters and cardiac output [169]. All the GH replacement trials showed that cardiac function returns to the pre-treatment setting upon cessation of GH treatment [158-160, 163, 164, 166, 169-172, 175].

The beneficial cardiovascular effects of GH replacement are related not only to cardiac anabolic actions but also to its peripheral effects. Treatment with GH normalizes NO production, thereby reducing peripheral vascular resistance and modulating cardiac cytoskeletal functions by altering calcium myofilament responsiveness [70, 157]. Moreover, GH replacement improves body composition, which is an important factor for reducing cardiovascular risk [176, 177], induces beneficial effects on lipid profile [178, 179] and reduces arterial intima-media thickness [152, 155, 178, 179].

## GH AND HEART FAILURE

The rationale for GH therapy in CHF appears evident when considering the cardiovascular effects of GH and the cardiac morphological and functional features in heart failure. Patients with CHF have reduced myocardial contractility, decreased cardiac output, dilated LV cavity, increased peripheral vascular resistance and enhanced wall stress. Cardiac dilatation, which initially helps to maintain an adequate stroke volume, initiates a vicious cycle whereby dilatation leads to dilatation. GH replacement may be beneficial in all steps of heart failure. By stimulating cardiac growth, GH induces a concentric pattern of remodelling, which reduces wall stress. By decreasing peripheral vascular resistance, GH reduces afterload, attenuates pathologic cardiac remodelling and improves cardiac function. Furthermore, by inducing positive inotropic effects, GH directly counteracts the impaired contractility, which is the *primum movens* of the vicious cycle responsible for pathologic remodelling.

The pathogenesis and the progression of CHF seem to be related also to an imbalance between pro-inflammatory/anti-inflammatory factors and endothelial dysfunction. Patients with CHF have excessive plasma levels of pro-inflammatory cytokines and impaired vascular reactivity, which consists of attenuated vasodilatation in response to acetylcholine and preserved response to the direct NO donor nitroprusside. By shifting the cytokine balance toward anti-inflammatory predominance and reducing pro-apoptotic factors, GH positively acts on LV remodelling, increasing LV contractile performance and enhancing exercise capacity. In addition, GH is able to improve vascular reactivity, not only by restoring NO production, but also activating non-endothelium-mediated actions, in particular by modifying intracellular calcium concentration and regulating Na^+^/K^+^ ATPase activity.

## EXPERIMENTAL STUDIES ON ANIMALS

The first study of the effects of the GH/IGF-1 system in experimental heart failure models dates back to 1992. At that time, Castagnino and colleagues evaluated the effect of GH on the connective tissue, fibroblast growth and proliferation in rats with experimental myocardial infarction, and found a significant decrease in the incidence of ventricular aneurysms [180]. A subsequent study, designed to assess the effects of IGF-1 on cardiac function and structure in rats with a doxorubicin-induced cardiomyopathy, showed that IGF-1 increases cardiac output as well as reduces histologically-detected myocyte damage [181]. In this scenario, Ito and co-workers proved, in cultured neonatal cardiomyocytes, that IGF-1, but not GH, promotes transcription of muscle-specific genes (namely, troponin I, myosin light chain-2, and α-actin), induces protein synthesis and increases myocyte size [75]. Duerr and colleagues demonstrated that IGF-1, administrated in rats early during the onset of experimental post-infarction heart failure, enhances the hypertrophic response of viable myocardium and cardiac performance [182]. Similarly, Cittadini and co-workers, investigating the cardiac effects of GH adminis-tration during the early phase of pathologic remodelling in a rat model of large myocardial infarction, confirmed that GH causes hypertrophy of the non-infarcted myocardium in a concentric pattern and improves LV function [183]. Two subsequent trials showed that GH plus IGF-1, given to rats with LV failure, starting one month after myocardial infarction, and then in the late phase of LV remodelling, improved cardiac function and reduced peripheral vascular resistance and LV dilatation [184, 185]. Other experimental studies confirmed that GH attenuates both the early and the late pathologic LV remodelling, induces hypertrophy of non-infarcted myocardium, improves LV function and increases cardiac output [186, 187].

Cittadini and co-workers administered GH or IGF-1 or GH plus IGF-1 to adult HF rats and found a significant increase in cardiac performance and LV mass, without development of significant fibrosis, and no additional hypertrophy in rats receiving GH plus IGF-1 compared with rats treated singularly with GH or IGF-1 alone. This interesting result suggested that, *in vivo*, IGF-1 mediates the GH-induced cardiac hypertrophy [188]. Subsequent studies confirmed that GH/IGF-1 modifies cardiac structure, reduces interstitial fibrosis and improves myocardial function [189-191].

More recently, Cittadini and colleagues demonstrated, in a rat model of post-infarction heart failure, that GH improves a broad spectrum of structural abnormalities of the extra-cellular matrix [187]. Specifically, they found a decrease in the collagen volume fraction and in the collagen I/III ratio, and an increase in capillary density. The authors hypothesized that GH attenuates fibrosis, directly by reducing collagen synthesis or increasing its breakdown, and indirectly by reducing accumulation of extracellular matrix proteins in the interstitial space. This latter was explained as due to the GH-induced improvement in hemodynamic and to the decrease in wall stress [187]. Cittadini and colleagues supposed that GH reduces interstitial fibrosis thanks also to its anti-apoptotic properties. Although apoptosis *per se* does not induce fibrosis, it leaves myocardial defects that are filled with interstitial fluid from myocardial edema, subsequently leading to fibrous tissue accumulation [187]. GH and IGF-1 exert direct beneficial effects on myocyte contractile performance in heart failure models, not solely by stimulating cardiac growth, modifying cardiac structure, reducing interstitial fibrosis or inducing peripheral vasodilatation, but also by changing calcium handling and the inotropic state [82, 88, 192-195]. Kinugawa and colleagues demonstrated that acute IGF-1 administration in isolated cardiomyocytes, in both normal and heart failure conditions, exerts a direct positive inotropic effect, due to calcium transient amplitude and calcium availability to the contractile apparatus [193]. They also showed IGF-1 does not modify the terminal portion of the relaxation phase trajectory, which indicates that calcium sensitivity is not altered by IGF-1 administration [193]. This result was consistent with previous studies in which acute IGF-1 administration increases the contractility of cardiomyocytes and isolated ventricular muscle [82, 88]. In addition, Freestone and colleagues reported that, in isolated rat cardiac muscle, acute IGF-1 administration had a positive inotropic effect, in fact, it increased the peak of cytosolic free calcium concentration, the amplitude of calcium transient and the time to peak [194]. In contrast with these results, Cittadini and co-workers showed that, in isolated isovolumic aequorin-loaded rat whole hearts and ferret papillary muscles, IGF-1 administration produces an acute positive inotropic effect, not associated with an increased intra-cellular calcium availability but to a significant increase of myofilament calcium sensitivity [82]. All these experimental studies, in which GH did not induce acute effects on cardiac function, and IGF-1 positively affected cardiac contractility, provide further insight into the intricate interaction between the GH/IGF-1 axis and cardiovascular system. In fact, although GHRs are expressed on the heart, their physio-logical role probably does not include acute modulation of myocardial contractility, but they serve to mediate such other functions as protein synthesis or local IGF-1 production [82, 88, 193, 194].

Von Lewinski and colleagues were the first to study the functional effects of IGF-1 in isolated human myocardium. They demonstrated that IGF-1: 1) exerts a concentration-dependent positive inotropic effect, which is almost completely prevented by blocking its receptors or phosphoinositide 3-kinase (PI3-kinase); 2) increases L-type calcium currents; 3) activates Na^+^-H^+^ and reversed Na^+^-Ca^2+^ exchanges [196]. The beneficial effects of GH treatment in heart failure may be also related to the anti-apoptotic proprieties of the GH/IGF-1 system [80, 81, 187, 197]. Although cardiomyocytes were long thought not undergo apoptosis, it is now recognized that cardiomyocyte apoptosis is increased in CHF and it may play a key role in CHF progression. Cardiomyocyte apoptosis occurs in the early stages of myocardial dysfunction; it impairs LV performance by reducing the contractile mass of the heart and by contributing to the progressive loss of myocytes [198, 199]. The anti-apoptotic effects of GH do not appear to be mediated by IGF-1: Gonzalez-Juanatey and co-workers demonstrated, in primary cultures of rat neonatal cardiomyocytes, that GH regulates apoptosis through the inhibition of calcineurin, a calcium-dependent phosphatase [197]. Others showed that the effects exerted by GH on cell survival and proliferation are mediated through two different signalling pathways, involving nuclear factor-kappa B (NF-kB) and PI3-kinase, respectively, which promote high circulating levels of the anti-apoptotic molecules [200-202].

## CLINICAL STUDIES IN HUMANS WITH HEART FAILURE

Several research groups have studied the effects of GH and IGF-1 in patients with impaired cardiac function (Table **1**). The first results were limited to case reports showing that GH administration considerably improved cardiac function [203, 204]. The earliest open clinical trial in CHF was reported by Fazio and co-workers in 1996 [90]. They studied seven patients with idiopathic dilated cardiomyopathy, with moderate to severe heart failure, without GHD. The evaluation was performed at baseline, after three months of recombinant  human  GH  (rhGH)  therapy  and three months after therapy discontinuation. They assessed cardiac function with Doppler echocardiography, right-heart catheterization and exercise testing. After three months of treatment at a dose of 4 international units every other day, they found improvement in cardiac performance, exercise tolerance, hemodynamic profile and myocardial energetic metabolism. Transthoracic echocardiography revealed a significant increase in relative wall thickness and cardiac mass, a dramatic decrease in wall stress and an improvement in systolic performance indices (ejection fraction, shortening velocity and aortic acceleration). Using right-heart catheterization to evaluate the effects of rhGH on hemodynamic variables, at rest and in response to physical exercise, they found significant decrease in mean pulmonary arterial and capillary wedge pressures, increased cardiac output and reduced systemic vascular resistance. The also demonstrated beneficial changes in myocardial energetic metabolism, particularly during physical exercise, i.e., the heart generated more mechanical work with lower oxygen consumption and energy production. This improvement in energetic metabolism was attributed to wall stress reduction and not to change in metabolic substrates. These encouraging preliminary findings prompted several larger and controlled clinical trials.

Conflicting results emerged from a randomized, double-blind, placebo-controlled rhGH treatment study, which showed, in fifty CHF patients, an increase in LV mass related to serum IGF-1 level but no change in LV wall stress, arterial blood pressure, ejection fraction, clinical status or 6-minute walking distance [205]. Similarly, in another clinical trial, carried out in twenty-two patients with CHF of various etiologies, rhGH treatment did not significantly affect clinical status, exercise duration, ejection fraction, end-diastolic and end-systolic volumes. Furthermore, no significant increases in LV mass and wall thickness were shown [206]. On the contrary, rhGH significantly increased exercise capacity and decreased LV end-systolic and end-diastolic volumes in patients with post-ischemic CHF [207]. The patients also had a 15% increase in posterior wall thickness and 16% increase in cardiac output [207]. rhGH did not affect cardiac structure but greatly improved exercise performance and quality of life in ten post-ischemic CHF patients [208]. Conflicting results were obtained from other numerous experimental trials. For instance, some studies showed that rhGH caused a significant increase in cardiac performance [209-211], whereas others found no changes [212-214].

More recently, Adamopoulos and colleagues investigated the immunomodulatory role of rhGH administration in CHF patients. They found that a three-month course of GH normalizes circulating levels of proinflammatory cytokines, such as tumour necrosis factor α (TNF-α) and interleukin- 6 (IL-6), their soluble receptors, as well as apoptosis mediators, such as soluble Fas (sFas) and soluble Fas ligand (sFasL) [215, 216]. They subsequently reported that GH reduces the soluble adhesion molecules ICAM-1 and VCAM-1, the granulocyte-macrophage colony-stimulating factor (GM-CSF), which generates free radicals and enhances cytokine production, and the macrophage chemoattractant protein-1 (MCP-1), which promotes the migration of mononuclear phagocytes into the injured myocardial tissue and endothelial cells [217]. To evaluate whether these changes are related to modifications in exercise tolerance and echocardiographic markers of cardiac remodelling and performance, they found a significantly correlation between improvement in exercise capacity and restoration to the normal of the inflammatory response, as well as a good correlation between exercise capacity improvement and reduction in adhesion molecules and in soluble apoptosis mediators. They also showed that GH induced a decrease in end systolic wall stress and an increase in contractile reserve and that these changes were correlated with the decrease in the chemotactic protein MCP-1 and pro-inflammatory cytokines [215-217].

**Fig. (2):**
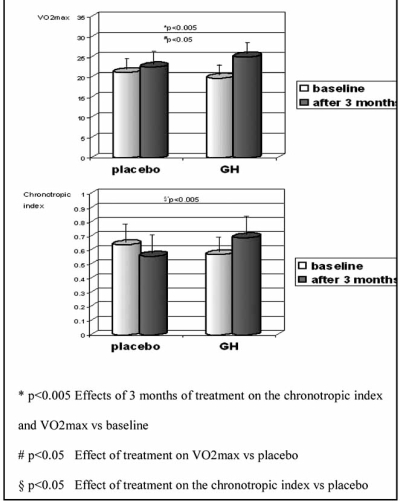
The effect of GH on VO2max and on the chronotropic index increased, and end-systolic wall stress and isovolumic relaxation time were reduced. Placebo did not affect any of the above parameters.

In an attempt to gain further insight into the mechanisms by which GH may benefit CHF patients, Fazio and co-workers have recently carried out a double-blind, placebo-controlled study of the effects of GH on physical exercise capacity and cardiopulmonary performance in twenty-two patients with moderate heart failure [218]. Patients underwent spirometry, cardiopulmonary exercise testing and Doppler echocardiography. The baseline clinical status was comparable in the GH patients and in the placebo group. After three months of treatment, at exercise testing, the GH group had an improvement of exercise capacity, cardio-pulmonary performance, and ventilatory efficiency, with a significant increase of VO2max and of chronotropic index (Fig. **2**).

Moreover, at transthoracic echocardiography, the GH group had an increase in LV mass index, relative wall thickness and cardiac performance. The LV ejection fraction and early-to-late mitral peak velocity ratio were significantly.

The conflicting results of the clinical trials of GH treatment analyzed in this review may be related to the small number of patients enrolled, the different dose and duration of GH treatment, the different CHF etiologies, and differences in the patients' demographic, hemodymamic and clinical characteristics. This discrepancy may also reflect the heterogeneity of IGF-1 increase in response to GH. In fact, a recent meta-analysis, which analyzed all randomized controlled trials and open studies on sustained GH treatment in adults with CHF in the absence of GHD, contained in the Medline, Biosis and EMBASE databases from their inception to June 2005, confirms that there is a close relationship between change in IGF-1 concentration and GH effects [219]. When the studies were divided into two groups based on the degree of IGF-1 increment, in trials with an IGF-1 increase >89% versus baseline there was a significant improvement in cardiac performance, echocardiographic parameters and exercise capacity, whereas in trials with an IGF-1 increase <89% there were no beneficial cardiovascular effects. In other words, patients with a blunted IGF-1 response to exogenous GH administration are less likely to benefit from GH treatment. This suggests that some patients may be not “sensitive” to GH. Therefore, “responders” should be identified before starting GH treatment in CHF patients.

## CONCLUSIONS

Although experimental models and preliminary human studies have demonstrated that GH administration may have beneficial cardiovascular effects in CHF, more experimental and clinical studies are necessary to clarify the mechanisms that determine the variable sensitivity to GH and its positive effects in the failing heart.

## Figures and Tables

**Table 1:** Characteristics of Clinical Studies on GH Treatment in Chronic Heart Failure

References	StudyDesign	Patients Enrolled	Age (mean ± SD)	Target dose (IU/wk)	IGF-1 Increase (%)	Therapy Duration (Months)	Outcomes
Fazio S_b_	Open	7	46 ± 9	14	105.1	3	HR, IVS, PW, LVM, EDD, ESD, ESWS, EF, E/A, IRT, SVR, ED, NYHA
Frustaci A_g_	Open	4	32 ± 8.1	28	NA	3	IVS, EDD, EF
Isgaard J_d_	Parallel	22	60 ± 11.3	0.25 IU/kg·wk up to 28	137.1	3	HR, IVS, PW, LVM, EDD, ESD, ESWS, EF, E/A, IRT, SVR, ED, NYHA
Osterziel K_c_	Parallel	50	54 ± 10	14	78.8	3	HR, IVS, PW, LVM, EDD, ESWS, EF, SVR, NYHA
Genth-Zotz S_e_	Open	7	55 ± 9	14	110.1	3	HR, PW, EDD, ESD, EF, SVR, VO_2_max, ED, NYHA
Jose VJ_h_	Open	6	NA	7	NA	6	IVS, PW, EDD, ESD, EF, ED, NYHA
Spallarossa P_f_	Parallel	20	62.1 ± 8	0.14 IU/kg·wk	89	6	IVS, PW, LVM, EDD, EF, E/A, IRT, ED, NYHA
Smit JW_k_	Parallel	22	65.5 ± 8.5	14	36.7	6	HR, LVM, EF, ESWS
Napoli R_a_	Parallel	16	54.5±11.3	14	85.5	3	HR, VO_2_max
Acevedo M_i_	Parallel	19	57.7 ± 4.5	0.245 IU/kg·wk	40.1	2	EF, VO_2_max
Adamopoulos S_m_	Cross- over	12	50 ± 13.8	14	NA	3	PW, ESWS, VO_2_max
Cittadini A_j_	Parallel	10	38.9±10.6	0.21 IU/kg·wk	NA	3	HR, IVS, PW, EF, E/A, SVR, ESWS
Fazio S_n_	Double-blind placebo controlled	22	PL:57±11 GH:54±10	14	101±18	3	MAP, VE, VO_2_max, RER, VE-VCOslope, Breathing reserve, chronotropic index, Mechanical workefficiency, CI, IRT, ESD, ESWS, EF, SVR, RWT

Adapted from: Le Corvoisier P, Hittinger L, Chanson P, Montagne O, Macquin-Mavier I, Maison P. Cardiac effects of growth hormone treatment in chronic heart failure: a meta-analysis. J Clin Endocrinol Metab 2007; 92: 180-5 (203).

CI, cardiac index; E/A, ratio between early and late mitral diastolic flow; ED, exercise duration; EDD, LV end-diastolic diameter; EF, ejection fraction; ESD, LV end-systolic diameter; ESWS, end-systolic wall stress; GH, growth hormone; HR, heart rate; IRT, isovolumetric relaxation time; IU, international unit; IVS, interventricular septum; kg, kilogram; LVM, LV mass; MAP, mean arterial pressure; NA, Not available; NYHA, New York Heart Association; PL, placebo; PW, posterior wall; RER, respiratory exchange ratio; RWT, relative wall thickness; SVR, systemic vascular resistance VE-VCO slope, minute ventilation-carbon dioxide production slope; VO2 max, maximal peak oxygen uptake; wk, week; a:[68]; b:[90]; c:[205];  d:[206];  e:[207];  f:[208]; g:[210]; h:[211]; i:[212]; j:[213]; k:[214]; m:[216]; n:[218].
